# Selective Production of Phenol-Rich Bio-Oil From Corn Straw Waste by Direct Microwave Pyrolysis Without Extra Catalyst

**DOI:** 10.3389/fchem.2021.700887

**Published:** 2021-07-01

**Authors:** Zhiyue Zhao, Zhiwei Jiang, Hong Xu, Kai Yan

**Affiliations:** Guangdong Provincial Key Laboratory of Environmental Pollution Control and Remediation Technology, School of Environmental Science and Engineering, Sun Yat-sen University, Guangzhou, China

**Keywords:** corn straw, microwave pyrolysis, temperature, power, phenol-rich bio-oil

## Abstract

We report a sustainable strategy to cleanly address biomass waste with high-value utilization. Phenol-rich bio-oil is selectively produced by direct pyrolysis of biomass waste corn straw (CS) without use of any catalyst in a microwave device. The effects of temperature and power on the yield and composition of pyrolysis products are investigated in detail. Under microwave irradiation, a very fast pyrolysis rate and bio-oil yield as high as 46.7 wt.% were obtained, which were competitive with most of the previous results. GC-MS analysis showed that temperature and power (heating rate) had great influences on the yield of bio-oil and the selectivity of phenolic compounds. The optimal selectivity of phenols in bio-oil was 49.4 area% by adjusting the operating parameters. Besides, we have made detailed statistics on the change trend of some components and different phenols in bio-oil and given the law and reason of their change with temperature and power. The *in situ* formed highly active biochar from CS with high content of potassium (1.34 wt.%) is responsible for the improvement of phenol-rich oils. This study offers a sustainable way to fully utilize biomass waste and promote the achievement of carbon neutrality.

## Introduction

In China, corn straw (CS) is the most abundant biomass waste, and its annual output has exceeded 216 million tons (Li et al., 2016a). How to valuably utilize this CS waste with minimum effect on the environment is crucial for peak carbon dioxide emissions before 2030 and achieving carbon neutrality before 2060. The conversion of biomass to high-value–added chemicals has attracted growing attention (Yi et al., 2020; Zhang et al., 2020). So far, gasification, pyrolysis, and liquefaction methods have been developed to deal with biomass waste (Hildebrandt et al., 2009; Vispute et al., 2010). However, biomass gasification is limited by high cost and low efficiency (Díaz González and Pacheco Sandoval, 2020). Furthermore, the liquid fuel from biomass liquefaction with low calorific value and more complex chemical composition needs further separation (Pang, 2019). Pyrolysis technology has been extensively exploited because it can convert biomass wastes from agricultural and forestry industry into liquid fuels (also called bio-oil) with wide adaptability (Liu et al., 2020; Al-Harahsheh et al., 2021; Hoang Pham et al., 2021). A number of highly valuable products can be separated from bio-oil. Among them, phenols in bio-oil are more environmentally friendly and have the potential to replace the phenolic compounds from the petroleum industry. Besides, they can be used as part of the phenolic resin instead of adhesive, disinfectant, developer, and so on.

Over the past few decades, efforts have been made to develop technologies for efficient pyrolysis of biomass and high product utilization systems (Durak, 2015; Durak, 2016; Durak et al., 2019). Microwave-assisted pyrolysis (MAP) with unique heating property is considered to be a very attractive technology for the production of high-value–added chemicals from biological residues (Pianroj et al., 2016; Bundhoo, 2018). Compared with traditional pyrolysis methods, MAP technology has some excellent properties, including fast heating rate (Borges et al., 2014), high yield of production, low oxygen content of bio-oil (Ferrera-Lorenzo et al., 2014a; Ravikumar et al., 2017), and volumetric heating (Mašek et al., 2013). Moreover, MAP can produce high-performance syngas, bio-oil (Kostas et al., 2020; Yu et al., 2020), and biochar (coal char) (Li et al., 2016b; Parvez et al., 2019). However, the bio-oil obtained by MAP of CS still contains considerable oxygen content (29.5 wt.%), which will inevitably lead to the generation of organic compounds with high oxygen contained in the pyrolysis organisms, among which phenols are the representative (Ferrera-Lorenzo et al., 2014b). For example, Dai et al. (Dai et al., 2020) prepared phenol-rich bio-oil by catalytic pyrolysis of corn cob with supported catalyst containing iron, and the phenol content reached 49.7 area% and the yield of bio-oil was 26.9 wt.%. By adjusting the composition of the catalyst and the reaction conditions, the selectivity reached 91.1 area%. Idris et al. (Idris et al., 2021) reported that the total phenolic compounds were 73.6 area% at low pyrolysis temperature (300°C) using activated carbon as a catalyst, but the yield of bio-oil was only 30.0 wt.%. Alisa et al. (Mamaeva et al., 2016) used activated carbon as a catalyst to convert raw biomass through MAP. By adjusting the proportion of catalysts and biomass and cracking conditions, the selectivity of total phenols reached 61.2 area% at 300°C, but the yield of bio-oil was less than 15.0 wt.%. Similarly, Joy et al. (Omoriyekomwan et al., 2016) used activated carbon for producing bio-oils from raw biomass materials. The selectivity of phenols reached 71.3 area%, and the conversion of bio-oil reached 36.8 wt.%. Albeit the previous works have made great progress, the following challenges are still faced: 1) extra catalyst is associated with the cost and the environmental issue in the synthesis; 2) the selectivity of phenols and yield of bio-oil failed to increase at the same time; and 3) the catalytic effect of biochar produced by microwave pyrolysis and alkali metal from biomass is often ignored.

In this work, we report the selective and green synthesis of phenol-rich bio-oils from CS waste by rapid pyrolysis without extra catalyst. This study will exhibit the following unique features: 1) No catalyst is used, avoiding the cost and the associated environmental issues in using organic solvents. 2) The *in situ* generated biochar formed in the process of pyrolysis and the alkali metal of biomass-self are found to play a crucial role in product distribution. 3) Through a series of characterization and adjustment of process parameters, the yields and selectivity of bio-oils are analyzed in detail. This work may offer a new way to clean processing of biomass waste.

## Experimental Section

### Raw Materials

The grain size of corn straw treated in Donghai County of Jiangsu Province in China ranged from 20 to 30 mesh. In each experiment, dichloromethane was mainly from Macklin Company with the purity of 99.5%. The microwave device was purchased from Changyi Microwave Company.

### Microwave Setup

**FIGURE 1:**
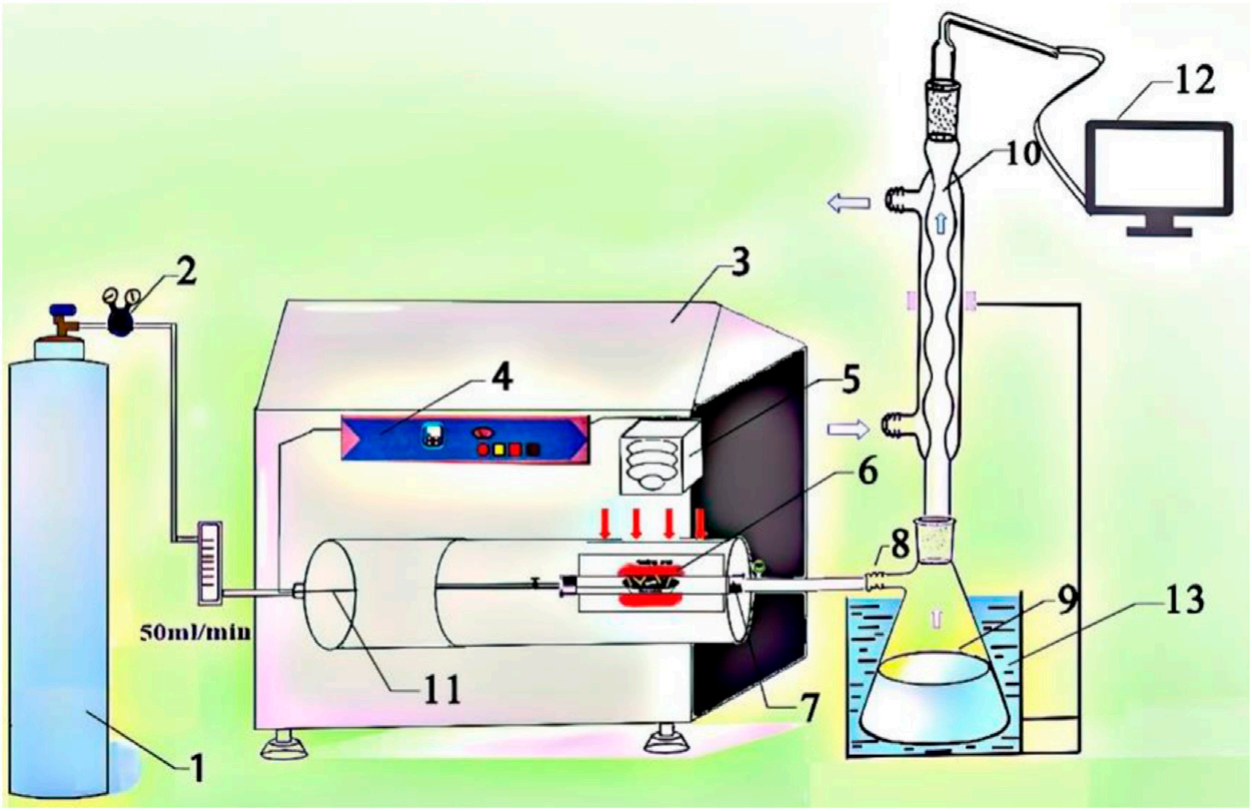
Schematic diagram of the microwave-assisted pyrolysis setup: (1) nitrogen cylinder; (2) cylinder valve; (3) microwave reaction equipment; (4) control panel; (5) thermocouple; (6) crucible (specific for microwave heating); (7) thermal insulation position; (8) rubber tube; (9) bio-oil collection bottle; (10) condensing tube; (11) temperature probe; (12) gas chromatograph; (13) cooling water.

The microwave pyrolysis experiment was carried out on a self-designed microwave device platform, as shown in Figure 1. The microwave device mainly consists of four parts: 1) the ventilation part is mainly composed of a nitrogen cylinder and cylinder valve; 2) the pyrolysis part is mainly completed in a microwave instrument, so it is mainly composed of a crucible (specific for microwave heating) and its surrounding semi-closed system; 3) the collection part is mainly composed of a bio-oil collection bottle and condensing tube; and 4) the gas collection part contains the air bag to collect the generated gas and GC analysis facility.

The first part is the carrier gas, the gas used in this work is high-purity nitrogen, and the ventilation rate is adjusted according to different purposes. The second part is pyrolysis reaction, which is mainly composed of a reaction device and raw materials. The reaction device here is mainly composed of a crucible (specific for microwave heating) and the surrounding semi-closed space. This is because the condensable gas produced by cracking reaction will have secondary cracking reaction in the surrounding semi-closed system, so the reaction part should be the whole quartz tube. The third part is the cooling device, which is mainly composed of three parts: the electric heating device in the microwave instrument, the condensing tube, and the water bath beaker. Condensation is an essential part of the pyrolysis reaction unit. Its condensation mode and condensation temperature have great influence on the yield and composition of bio-oil. The first-stage condensing device in this experiment is at thermal insulation position in the microwave equipment shown in Figure 1. The temperature of this position is controlled by a control panel. We set the temperature of heat preservation at 200°C. The secondary condensing device is the conventional condensation of tap water on the condensing tube and the water bath condensation formed by the conical flask and the beaker filled with water. We add dichloromethane into the conical flask in advance to facilitate the absorption of volatile matter. The condensation device and condensation temperature in the laboratory are universal, which have guiding significance for industrial production and other scientific research work. Too high or too low condensation temperature will lead to insufficient utilization of biomass pyrolysis resources. Therefore, we adopt a self-designed condensation mode to collect phenol-rich bio-oil, which provides the basis for other related work.

### MAP Experiments

CS (1.50 ± 0.05 g) was placed in a crucible that is specially made for microwave heating to absorb microwaves. The crucible was put into a quartz tube, where it was pushed to the middle position. When the reaction temperature was below 150°C, the ventilation rate was set as 150 ml/min. It then reduced to 50 ml/min when increasing the temperature above 150°C. Taking 100°C as the interval and 400, 500, 600, and 700°C as the reaction conditions, the effects of temperature on the yield and different components of bio-oil were studied. When doing the experiment of temperature influencing factors, we set the power to the maximum of 1300 W, raise it to the target temperature at the fastest rate, and keep it for 20 min, so as to reduce the influence of power on temperature. Under the reaction conditions of 600, 800, 1000, and 1200 W, the effect of power on the yield and composition of bio-oil was explored, and each experiment lasted for 20 min. The reaction time is determined according to the principle that there is no phenomenon at the end of pyrolysis reaction.

After the pyrolysis reaction is completed, there is a small amount of water in the connection between the reaction device and the cooling device, and part of the bio-oil is found in the six places in Figure 1. The specific value of the residue was calculated by weighing the mass of the device (6, 7, 8, and 9) before and after the reaction, and the error was within ±1%. Finally, we explored the composition and distribution of bio-oil in the quartz wall around the crucible, thermal insulation position, rubber tube, and bio-oil collection bottle, especially in the analysis of bio-oil in the rubber tube and bio-oil collection bottle. The yield of pyrolysis products was obtained by the following formulas:

$$\;{\text{Y}}_{\text{bio}-\text{oil}}\;=\frac{M_{oil}}{Mi}\;\;\times \;100\text{\%,}$$ (1)

$${\text{Y}}_{\text{biochar}}\;=\;\frac{M_{biochar}}{Mi}\;\times \;100\text{\%,}$$ (2)

$${\text{Y}}_{\text{gas}}{\;=1‐\text{ Y}}_{\text{bio}-\text{oil}}{\;‐\text{ Y}}_{\text{biochar}},$$ (3)

where Y_bio-oil_ represents the yield of bio-oil, Y_biochar_ represents the yield of biochar, and Y_gas_ represents the yield of syngas.

### Measurement and Analysis

The metal elements of corn straw before and after pyrolysis were detected on an Agilent 5000 inductively coupled plasma-optical emission spectrometer (ICP-OES), and the chlorine elements were detected by a halogen analyzer (LC-2010plus). The thermal behavior analysis of CS was studied on an STA449F3 thermal analyzer (Netzsch, Germany) heating from 30 to 800°C with different heating rates. In a typical experiment, about 5 mg CS powder was introduced into the crucible and heated in N_2_ from 30 to 800°C. N_2_ was used as a protective gas, and the flow rate was 50 ml/min.

The chemical compositions of bio-oil were detected using a gas chromatography–mass spectrometry system (DSQII, Thermo Fisher, United States) equipped with a TG-5 SilMS capillary column. High-purity (99.999%) helium was used as the carrier gas, and the flow rate was 1 ml/min. The injection size was 0.2 μL with the split ratio of 1:20. The initial temperature of the column temperature chamber was 40°C and maintained for 3 min, which then raised to 320°C at a heating rate of 10°C/min for 5 min. The injection port temperature was 250°C, the ion source temperature was 230°C, and the interface temperature was 280 °C. The scanning range of MS was 19–500°m/z, and the scanning speed was 2009.4. The final results were compared with the National Institute of Standards and Technology (NIST-2.0) mass spectrometry database to identify the compounds in the bio-oil.

## Results and Discussion

### TG-DTG Analysis of CS

**FIGURE 2:**
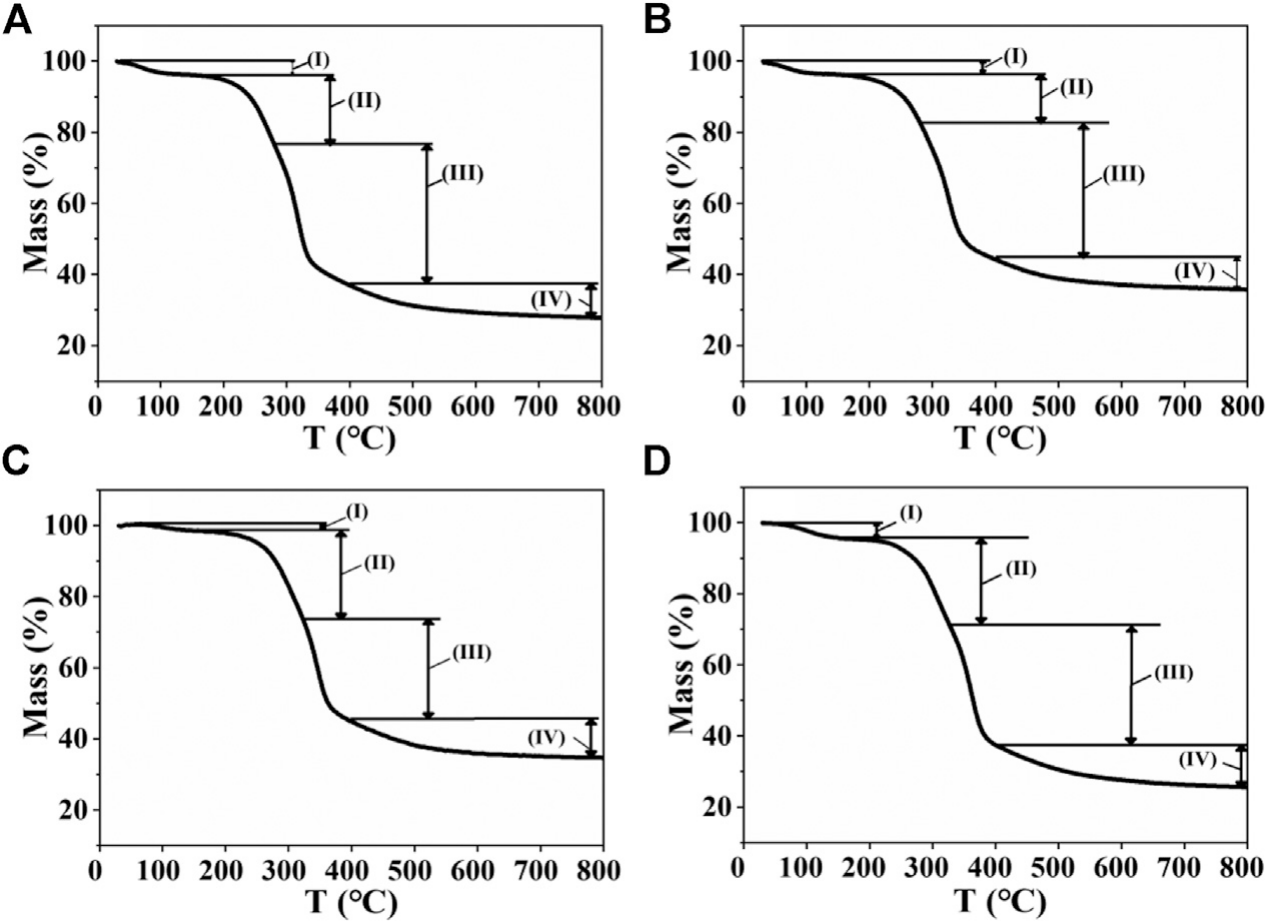
Thermogravimetric curves at different heating rates of **(A)** 5°C/min, **(B)** 10°C/min, **(C)** 30°C/min, and **(D)** 50°C/min.

The thermal degradation of corn straw with different heating rates was examined by thermogravimetry (TG), as shown in Figure 2. The related weight loss rate in the TG curves is listed in Table 1. The TG curves can be divided into four stages: 1) The physisorbed water was completely evaporated at 30∼170°C. The total weight loss of the drying stage at 5, 10, 30, and 50°C/min was 3.4 wt.%, 3.5 wt.%, 1.8 wt.%, and 3.7 wt.%, respectively. 2) Hemicellulose and lignin in CS were degraded at 170∼330°C with different heating rates. The weight loss rate of 20.7, 21.0, 24.6, and 26.2 wt.% was achieved at different heating rates of 5, 10, 30, and 50°C/min, respectively, revealing that high heating rate could improve the weight loss in this stage. 3) Cellulose and lignin in CS were degraded at 280∼400°C with different heating rates, and the weight loss in this stage is very obvious. In this stage, the weight loss with different heating rates was relatively stable (about 35 wt.%). The weight loss at different heating rates of 5, 10, 30, and 50°C/min was 38.2, 30.7, 29.0, and 31.5 wt.% (Table 1), respectively. 4) The residue was continuously pyrolyzed at 400∼800°C. In this stage, slow weight loss occurred because of the slow carbonization of residual materials. The above results revealed that high heating rate could improve hemicellulose pyrolysis. The heating rate also affected the results of the total weight loss. The total weight loss corresponding to the four heating rates is 72.2, 64.3, 65.4, and 74.1 wt.%, respectively.

**TABLE 1:** Weight loss rate of corn straw in different stages with different heating rates.

Heating rate (°C/min)	Stage loss rate (wt.%)				Total weight loss (wt.%)
	State (I)	State (II)	State (III)	State (IV)	
	TR	TR	TR	TR	
5°C/min	4.1%	20.8%	38.2%	9.1%	72.2
	30–166°C	166–283°C	283–400°C	400–800°C	
10°C/min	4.1%	21.0%	30.7%	8.5%	64.3
	30–163°C	163–284°C	284–400°C	400–800°C	
30°C/min	1.5%	24.6%	29.0%	10.3%	65.4
	30–160°C	160–322°C	322–400°C	400–800°C	
50°C/min	4.5%	26.2%	31.5%	11.9%	74.1
	30–158°C	158–331°C	331–400°C	400–800°C	

TR, temperature range.

**FIGURE 3:**
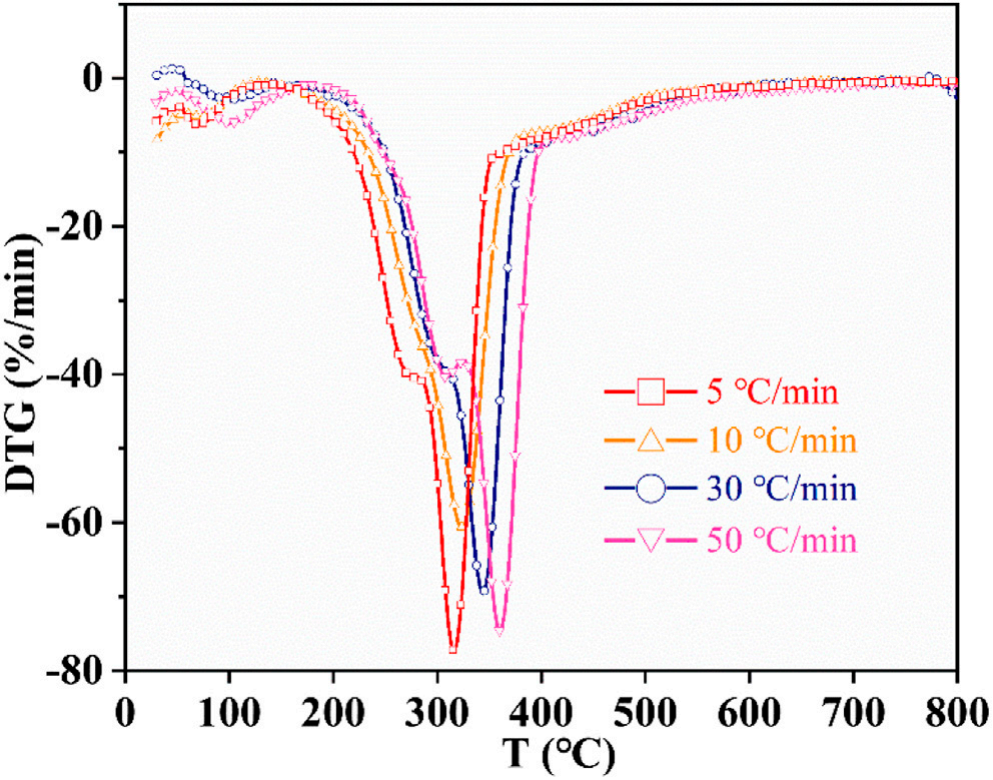
DTG curves of corn straw under different heating rates in nitrogen atmosphere.

Figure 3 shows the DTG curves of CS with different heating rates. In particular, the shoulder peaks in the DTG curve at 318∼362°C were assigned to the degradation of hemicellulose, and the sharp peaks at 354∼398°C were caused by the degradation of cellulose. Furthermore, the shoulder peaks and sharp peaks shifted to high temperature with increasing heating rates from 5 to 50°C/min. This may be because a slow heating rate can prolong the residence time of the sample particles, thus promoting the degradation and carbonization of cellulose and hemicellulose at a lower temperature.

### Effect of Different Conditions on the Bio-Oil Yield

**FIGURE 4:**
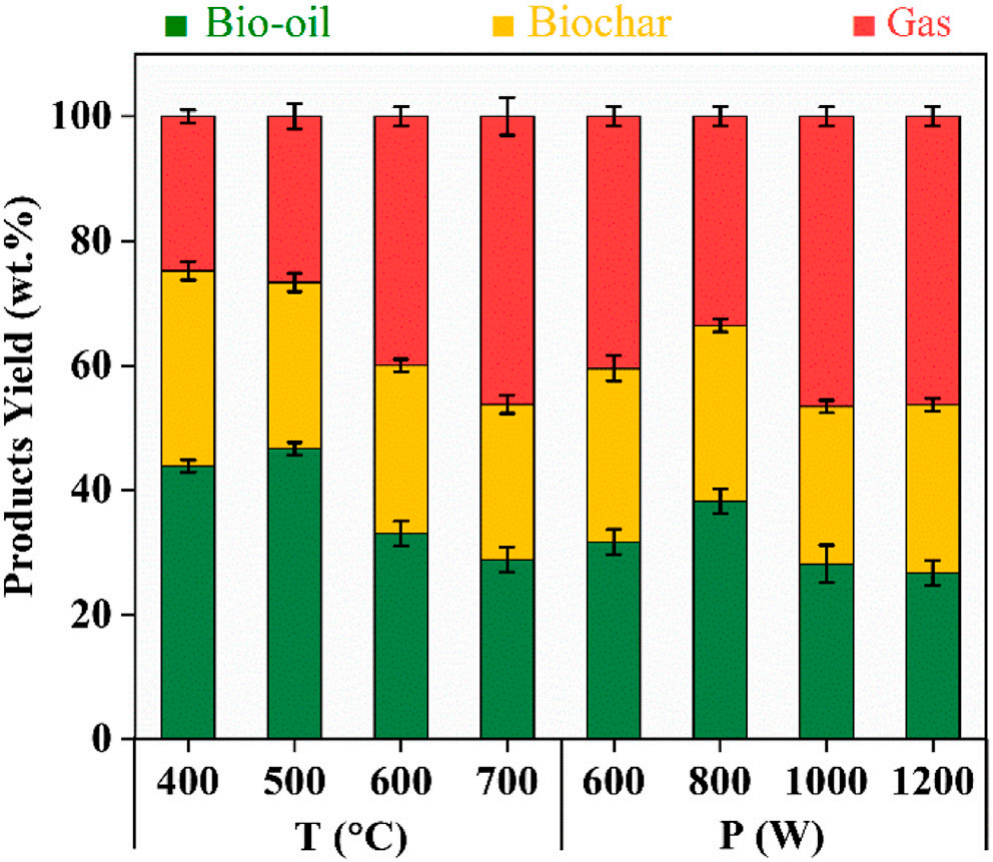
Distribution of pyrolysis products from corn straw at different temperatures and power.

The yields of three products (bio-oil, biochar, and gas) corresponding to different temperatures and power are shown in Figure 4. The reaction time for CS through MAP at different temperatures is 20 min. With the increase of temperature, the yield of bio-oil increased from 43.8 wt.% at 400°C to a maximum value of 46.7 wt.% at 500°C. In contrast, the yield of syngas decreased from 24.8 to 22.0 wt.% and the yield of biochar basically remained unchanged (about 31 wt.%). Further increasing the reaction temperature from 500 to 700°C, the yields of bio-oil and biochar sharply reduced and the yield of syngas significantly improved. This may be attributed to the further decomposition of the generated bio-oil or biochar, leading to the generation of syngas. Bio-oil was mainly derived from the pyrolysis of cellulose and hemicellulose, while biochar was from lignin (Kumar et al., 2020). Furthermore, the pyrolysis temperatures of the three components in biomass (hemicellulose, cellulose, and lignin) were 200–300°C (Kong et al., 2014), 247–427°C (Ding et al., 2020), and 205–500°C (Chen and Kuo, 2010), respectively. This result showed that increasing temperature is conducive to the pyrolysis reaction and improving the yield of bio-oil at temperatures below 500°C. Further increasing the temperature caused the reduction of bio-oil yield. Therefore, the optimal operation temperature for the generation of bio-oil from CS pyrolysis is 500°C.

The effect of power ranging from 600 to 1200 W on the yield of bio-oil from CS was studied. The reaction time for CS through MAP under different power is 40 min. Similarly, with the increase of power, the yield of bio-oil increases first and then decreases. The maximum yield of bio-oil is around 38.1 wt.% under 800 W, which is lower than that achieved at reaction temperature 500°C. This can be explained by that long reaction time could have resulted in the degradation of the obtained bio-oil. Further increasing the operation power resulted in a lower yield of bio-oil and a higher yield of syngas. This could be because a high power caused a high reaction temperature (above 500°C) and thus reduced the yield of bio-oil.

### Effects of Different Conditions on Bio-Oil Components

**FIGURE 5:**
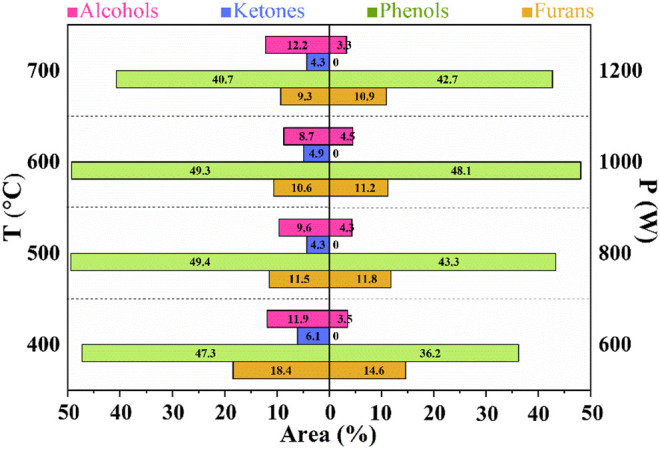
Distribution of main components of bio-oil by microwave pyrolysis of CS at different temperatures and power.

GC-MS was used to explore the component distribution of bio-oil under different reaction temperatures and power. The distribution of main components in bio-oil obtained by microwave pyrolysis of CS at different temperatures and power is shown in Figure 5. The partial analysis results of the bio-oils are also listed in Tables 2, 3. The analysis results of other conditions are shown in the supplementar1111111111y information (Supplementary Tables S1–S6). In addition, the corresponding GC-MS chromatograms under different conditions are shown in Supplementary Figures S1, S2.

**TABLE 2:** Relative proportion (area%) of most components of CS-based bio-oil at 500°C.

Entry	RT (min)	Peak name	Area%
1	4.22	3-Hydroxy-1,8-nonadiene	0.8
2	5.5	3-Furaldehyde	0.8
3	5.96	2-Furanmethanol	2.4
4	6.22	1-Acetoxyacetone	2.0
5	6.95	2-Methylcyclopentenone	0.8
6	7.03	2,4-Dimethylcyclohexan-1-ol	2.7
7	8.07	4-Ethyl-4-methylcyclohex-2-en-1-one	1.6
8	8.42	Phenol	8.7
9	8.81	H-Gly-DL-Thr-OH	0.9
10	9.16	Methyl cyclopentenolone	3.5
11	9.7	*o*-Cresol	3.1
12	10.06	*p*-Cresol	6.2
13	10.24	Guaiacol	5.0
14	10.71	3-Ethyl-2-hydroxy-2-cyclopenten-1-one	1.9
15	11.51	4-Ethylphenol	6.1
16	11.89	Creosol	1.7
17	12.32	2,3-Dihydrobenzofuran	8.3
18	13.14	4-Ethyl-2-methoxyphenol	3.0
19	13.67	4-Hydroxy-3-methoxystyrene	6.2
20	14.16	Syringol	5.4
21	15.53	*trans*-Isoeugenol	1.0
22	16.02	3-Hydroxydodecanoic acid	2.5
23	16.12	5-*tert*-Butylbenzene-1,2,3-triol	1.1
24	18.52	2,6-Dimethoxy-4-allylphenol	2.0
25	18.81	3,7,11-Trimethyldodecan-1-ol	0.7

RT, retention time.

**TABLE 3:** Relative proportion (area%) of most components of corn stalk–based bio-oil at 1000 W.

Entry	RT (min)	Peak name	Area%
1	6.05	3-Furancarbinol	1.8
2	8.47	Phenol	5.7
3	9.21	Methyl cyclopentenolone	2.6
4	9.75	*o*-Cresol	3.8
5	10.11	*p*-Cresol	4.6
6	10.29	Guaiacol	5.3
7	10.77	3-Ethyl-2-hydroxy-2-cyclopenten-1-one	1.9
8	11.56	4-Ethylphenol	6.3
9	11.94	Creosol	1.8
10	12.37	2,3-Dihydrobenzofuran	9.3
11	13.20	4-Ethyl-2-methoxyphenol	3.7
12	13.72	4-Hydroxy-3-methoxystyrene	7.3
13	14.20	Syringol	5.2
14	16.02	2,6,10-Trimethyltetradecane	1.8
15	16.27	2,4-Di-*tert*-butylphenol	1.4
16	16.44	5-*tert*-Butylbenzene-1,2,3-triol	1.2
17	18.57	2,6,10-Trimethyltetradecane	3.2

RT, retention time.

Ketones, phenols, acids, alcohols, aldehydes, esters, furans, and hydrocarbons in the bio-oil can be obtained from CS in our work, which is consistent with results from other groups (Kumar et al., 2020; Pang et al., 2020). The effect of temperature on the component in bio-oil was studied. With the increase of temperature, the total phenol content first increased and then decreased. The highest content of phenols was 49.4 area% at 500°C. After 500°C, the content of phenolic compounds decreased, which is consistent with the results reported by Lou et al. (Lou et al., 2010), and the lowest content of phenols was 40.7 area% at 700°C. As we all know, phenols are derived from lignin in CS and the decomposition temperature of lignin is below 500°C. The high content of phenols at 500°C could be explained by that lignin was cracked thoroughly and converted into phenols. Further increasing the temperature (above 500°C) improved the degradation of the obtained phenols and caused a decrease in the phenol content. The result verified that the reaction temperature could make great impacts on the phenol content in the obtained bio-oil. As the reaction temperature increased from 400 to 700°C, the furan content gradually decreased from 18.4 area% to 9.3 area%, indicating that the furans can be converted into other products at a relatively high temperature. The alcohol content first reduced from 11.9 area% at 400°C to 7.3 area% at 600°C and then increased to 12.2 area% at 800°C, confirming that alcohols may be produced from phenols or furans. The ketone content in the whole reaction temperature had basically no change (about 5.4 area%).

Different power from 800 to 1200 W was carried out for the microwave pyrolysis of CS. Similarly, with the increase of power, the yield of phenol content firstly enhanced (below 1000 W) and then decreased (above 1000 W). The highest content of phenols was 48.1 area% under 1000 W. As the reaction power increased from 600 to 1200 W, the furan content gradually decreased from 14.6 area% to 10.9 area%, indicating that the furans can be converted into other products with the increase of power. The content of alcohols at different power basically remained unchanged (about 4 area%), which was lower than that obtained at different temperatures. Furthermore, no ketones can be achieved for CS through MAP under different power. The reaction time at different temperatures (20 min) was less than that under different power (40 min). Therefore, long reaction time could cause degradation of alcohols as well as ketones and decrease their contents.

### Effects of Different Conditions on the Content of Phenols

The pathways of lignin decomposition for the production of phenols are generally divided into the following categories: (I) phenol, (II) alkylphenols, (III) alkoxyphenols, (IV) syringol, and (V) guaiacol. Because guaiacol and syringol belong to alkoxyphenols, only three kinds of phenol can be produced from lignin. However, guaiacol and syringol are widely used as research objects because of their wide application range and high added value.

**FIGURE 6:**
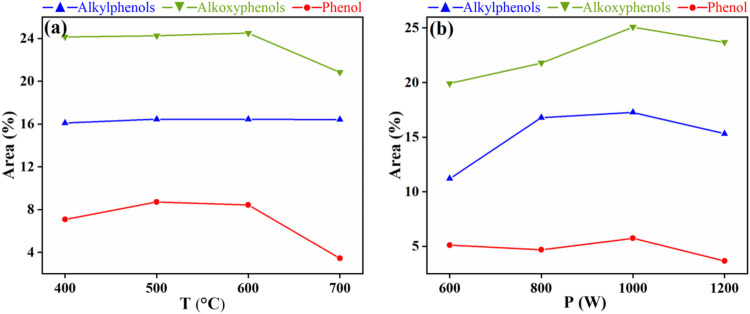
Effects of temperature **(A)** and power **(B)** on relative contents of different phenols.

**FIGURE 7:**
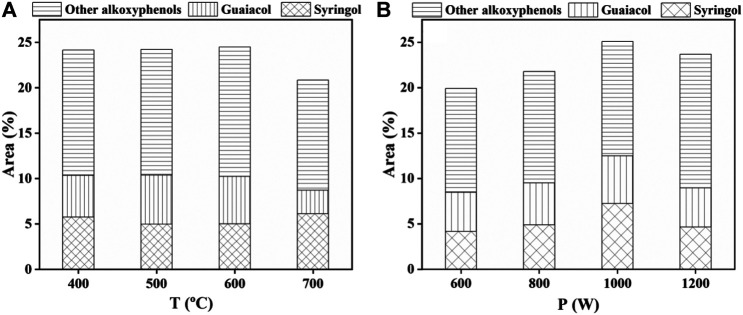
Distribution of alkoxyphenols in bio-oil obtained at different **(A)** temperatures and **(B)** power.

As shown in Figure 6, the relative content of the three phenols was decreased in the following order: alkoxyphenols (including syringol and guaiacol) > alkylphenols > phenol, at different temperatures or power. With the increase of temperature, the relative content of alkoxyphenols first increased to a maximum of 24.5 area% at 600°C and then decreased at 700°C, indicating that a higher temperature leads to the degradation of alkoxyphenols. The relative content of alkylphenols (about 16.4 area%) was unchanged, confirming a well stability of alkylphenols in the temperature range between 400 and 700°C. The relative content of phenol basically stayed invariable (about 8 area%) from 400 to 600°C and then decreased to 3.4 area% at 700°C. With the increase of power, the relative content of alkoxyphenols first increased from 19.9 area% under 600 W to 25.1 area% under 1000 W and then decreased slightly to 23.7 area% under 1200 W. The relative content of alkylphenols first increased to a maximum of 17.3 area% under 1000 W and then decreased. The average content of phenol under different power was about 4.5 area%. The above results revealed that a higher temperature (above 600°C) caused the degradation of alkoxyphenols and phenol and a higher power (above 1000 W) caused the degradation of alkoxyphenols and alkylphenols. The distribution of eugenol, guaiacol, and other alkoxyphenols under different conditions was also analyzed and is shown in Figure 7. The average contents of eugenol and guaiacol were 4.8 area% and 5.1 area% under different temperature conditions and 4.7 area% and 5.6 area% under different power conditions.

### Comparison of Alkali Metals in the Pyrolysis Process

Alkali metals, alkaline earth metals (Lv et al., 2010), and chlorine (Tang et al., 2014) could significantly improve the bio-oil yield and enhance the phenol selectivity in bio-oil during biomass pyrolysis. Therefore, the metal content of corn straw before and after pyrolysis was determined by the ICP method, and the chlorine content of corn straw before and after pyrolysis was determined by a halogen analyzer. The results are listed in Table 4. It was obvious that the content of K and Cl elements in corn straw is much more than that of other metal elements (Na, Mn, Fe, Ni, and Ce). After pyrolysis, the contents of K and Cl in corn straw increased sharply from 1.34 wt.% and 0.79 wt.% to 4.67 wt.% and 2.82 wt.%, respectively. As a result, the high yield of bio-oil and high selectivity of phenols could possibly be related to the high content of K and Cl.

**TABLE 4:** Element composition of corn straw before and after pyrolysis.

Elements	Before pyrolysis (wt.%)	After pyrolysis (wt.%)
K	1.34%	4.67%
Na	0.02%	0.06%
Mn	0.08%	0.03%
Fe	0.02%	0.07%
Ni	<0.01%	<0.01%
Ce	<0.01%	<0.01%
Cl	0.79%	2.82%

Compared with the work of other researchers, the selectivity of phenol content in this work is particularly high. The possible reasons are as follows: 1) Cellulose or hemicellulose can promote the pyrolysis of lignin to produce more phenolic compounds (Sun et al., 2020). 2) The microwave device is suitable for the production of phenolic substances because the first-stage condensation temperature of this work is higher. When the first-stage condensation temperature is higher than 100°C, the proportion of the lignin pyrolysis oligomer in bio-oil is increasing (Sui et al., 2014). 3) Biochar formed in the process of microwave pyrolysis worked as an auto-catalyst (microwave absorbent) forming a hot spot effect. These hot spots can generate hydrogen under the action of microwaves (Bu et al., 2013). Hydrogen contributes to the degradation of lignin in CS and promotes the deoxidation of these degraded fragments. After a series of decarboxylation and dehydration, the selectivity of phenolic components is improved. 4) Alkali metals from biomass-self would promote the production of phenol-rich oils.

## Conclusion

We have successfully produced phenol-rich bio-oil by the direct pyrolysis process of CS without use of any catalyst in a microwave device. The highest bio-oil yield (46.7 wt.%) was obtained at 500°C, and the highest bio-oil yield was 38.2 wt.% at 800 W. Besides, we have thoroughly explored some characteristic parameters (e.g., pyrolysis temperature and power of microwaves) and analyzed the weight loss rate as well as degree at various stages of different pyrolysis processes. This work offers the following features: 1) We developed a facile procedure for high value utilization of biomass waste without extra catalyst, avoiding the additional cost and environmental issues in the synthesis of the catalyst. 2) Over 46.7 wt.% yield of bio-oil yield at a low temperature of 500°C and 38.2 wt.% yield at 800 W power were obtained, which are the highest values in comparison with those of previous studies. 3) The alkali metal from CS and the *in situ* generated biochar worked as self-catalysts (microwave absorbents) to enhance the yields of phenols. 4) The effects of different power and temperatures on various kinds of oxygenated organic compounds were studied, and a scheme for producing high yield of bio-oil by microwave pyrolysis without catalyst was provided. The methodology developed in this work may cater for microwave pyrolysis of various biomass wastes to produce phenol-rich bio-oils.

## Data Availability

The raw data supporting the conclusions of this article will be made available by the authors, without undue reservation.
