# Supplementary material for: Selective Production of Phenol-Rich Bio-Oil From Corn Straw Waste by Direct Microwave Pyrolysis Without Extra Catalyst
Source: Front Chem. 2021 Jul 1;9:700887. doi: 10.3389/fchem.2021.700887 (PMC8280759; doi:10.3389/fchem.2021.700887)
Supplement: Supplementary file 1 [file DataSheet1.docx]

**SUPPLEMENTARY DATA**

**Selective production of phenols-rich bio-oil from corn straw waste direct by microwave pyrolysis without extra catalyst**

Zhiyue Zhao, Zhiwei Jiang, Hong Xu, Kai Yan*

*Guangdong Provincial Key Laboratory of Environmental Pollution Control and Remediation Technology, School of Environmental Science and Engineering, Sun Yat-sen University, Guangzhou 510275, China*

^*^Corresponding author *E-mail address*: yank9@mail.sysu.edu.cn (K. Yan).

| **Content** | **Subjects** | **Pages** |
| --- | --- | --- |
| Table S1 | Relative proportion (area%) of most components of corn stalk based bio-oil at 400 °C. | 2 |
| Table S2 | Relative proportion (area%) of most components of corn stalk based bio-oil at 600 °C. | 3 |
| Table S3 | Relative proportion (area%) of most components of corn stalk based bio-oil at 700 °C. | 4 |
| Table S4 | Relative proportion (area%) of most components of corn stalk based bio-oil at 600 W. | 5 |
| Table S5 | Relative proportion (area%) of most components of corn stalk based bio-oil at 800 W. | 6 |
| Table S6 | Relative proportion (area%) of most components of corn stalk based bio-oil at 1200 W. | 7 |
| Figure S1 | GC-MS chromatogram of bio-oil produced by microwave pyrolysis of CS at (a)400 ℃, (b) 500 ℃, (c) 600 ℃, (d) 700 ℃. | 8 |
| Figure S2 | GC-MS chromatogram of bio-oil produced by microwave pyrolysis of CS at (a) 600 W, (b) 800 W, (c) 1000 W, (d) 1200 W. | 9 |

**Table S1.** Relative proportion (area%) of most components of corn stalk based bio-oil at 400 °C.

| **Entry** | **RT(min)** | **Peak name** | **Area%** |
| --- | --- | --- | --- |
| 1 | 4.22 | 3-Hydroxy-1,8-nonadiene | 1.6 |
| 3 | 5.50 | 3-Furaldehyde | 6.1 |
| 4 | 5.96 | 2-Furanmethanol | 3.2 |
| 5 | 6.22 | 1-Acetoxyacetone | 2.4 |
| 6 | 6.95 | 2-Methylcyclopentenone | 1.1 |
| 7 | 7.03 | 2,4-Dimethylcyclohexan-1-ol | 3.2 |
| 8 | 8.07 | 4-Ethyl-4-methylcyclohex-2-en-1-one | 2.6 |
| 9 | 8.42 | Phenol | 7.1 |
| 10 | 8.81 | H-Gly-DL-Thr-OH | 1.1 |
| 11 | 9.16 | Methyl cyclopentenolone | 4.6 |
| 12 | 9.70 | O-Cresol | 3.0 |
| 13 | 10.06 | p-Cresol | 5.5 |
| 14 | 10.24 | Guaiacol | 5.8 |
| 15 | 10.71 | 3-Ethyl-2-hydroxy-2-cyclopenten-1-one | 1.8 |
| 16 | 11.51 | 4-Ethylphenol | 6.9 |
| 17 | 11.89 | Creosol | 1.5 |
| 18 | 12.32 | 2,3-Dihydrobenzofuran | 9.1 |
| 19 | 13.14 | 4-Ethyl-2-methoxyphenol | 3.1 |
| 20 | 13.67 | 4-Hydroxy-3-methoxystyrene | 6.8 |
| 21 | 14.16 | Syringol | 4.6 |
| 22 | 15.53 | Trans-isoeugenol | 0.8 |
| 23 | 16.02 | 3-Hydroxydodecanoic acid | 1.8 |
| 24 | 16.12 | 5-Tert-butylbenzene-1,2,3-triol | 0.7 |
| 25 | 18.52 | 2,6-Dimethoxy-4-allylphenol | 1.6 |
| 26 | 18.81 | 3,7,11-Trimethyldodecan-1-ol | 0.8 |

RT: retention time.

**Table S2.** Relative proportion (area%) of most components of corn stalk based bio-oil at 600 °C.

| **Entry** | **RT (min)** | **Peak name** | **Area%** |
| --- | --- | --- | --- |
| 1 | 5.49 | 3-Furaldehyde | 0.8 |
| 3 | 5.97 | 2-Furanmethanol | 2.3 |
| 4 | 6.22 | 1-Acetoxyacetone | 2.6 |
| 5 | 6.95 | 2-Methylcyclopentenone | 0.7 |
| 6 | 7.04 | 2,4-dimethylcyclohexan-1-ol | 2.6 |
| 7 | 8.09 | 4-ethyl-4-methylcyclohex-2-en-1-one | 1.6 |
| 8 | 8.42 | Phenol | 8.4 |
| 9 | 8.81 | H-Gly-DL-Thr-OH | 0.9 |
| 10 | 9.16 | Methyl cyclopentenolone | 3.4 |
| 11 | 9.70 | O-Cresol | 3.0 |
| 12 | 10.06 | p-Cresol | 6.2 |
| 13 | 10.24 | Guaiacol | 5.0 |
| 14 | 10.71 | 3-Ethyl-2-hydroxy-2-cyclopenten-1-one | 1.9 |
| 15 | 11.51 | 4-Ethylphenol | 6.1 |
| 16 | 11.88 | Creosol | 1.8 |
| 17 | 12.31 | 2,3-Dihydrobenzofuran | 8.8 |
| 18 | 13.14 | 4-Ethyl-2-methoxyphenol | 3.1 |
| 19 | 13.67 | 4-Hydroxy-3-methoxystyrene | 6.3 |
| 20 | 14.15 | Syringol | 5.2 |
| 21 | 15.53 | Trans-isoeugenol | 1.1 |
| 22 | 16.02 | 3-Hydroxydodecanoic acid | 2.6 |
| 23 | 16.40 | 5-Tert-butylbenzene-1,2,3-triol | 1.1 |
| 24 | 18.52 | 2,6-Dimethoxy-4-allylphenol | 2.0 |
| 25 | 18.81 | 3,7,11-trimethyldodecan-1-ol | 0.7 |

RT: retention time.

**Table S3.** Relative proportion (area%) of most components of corn stalk based bio-oil at 700 °C.

| **Entry** | **RT (min)** | **Peak name** | **Area%** |
| --- | --- | --- | --- |
| 1 | 4.22 | 3-Hydroxy-1,8-nonadiene | 1.2 |
| 2 | 4.65 | 1,3-Cyclopentanediol | 0.6 |
| 3 | 5.50 | 3-Furaldehyde | 0.8 |
| 4 | 5.97 | 2-Furanmethanol | 2.3 |
| 5 | 6.22 | 1-Acetoxyacetone | 2.6 |
| 6 | 6.95 | 2-Methylcyclopentenone | 0.7 |
| 7 | 7.04 | 2,4-dimethylcyclohexan-1-ol | 2.6 |
| 8 | 8.09 | 4-ethyl-4-methylcyclohex-2-en-1-one | 0.9 |
| 9 | 8.42 | Phenol | 3.4 |
| 10 | 8.73 | H-Gly-DL-Thr-OH | 3.0 |
| 11 | 9.16 | Methyl cyclopentenolone | 6.2 |
| 12 | 9.70 | O-Cresol | 5.0 |
| 13 | 10.06 | p-Cresol | 1.9 |
| 14 | 10.24 | Guaiacol | 6.1 |
| 15 | 10.71 | 3-Ethyl-2-hydroxy-2-cyclopenten-1-one | 1.8 |
| 16 | 11.51 | 4-Ethylphenol | 8.8 |
| 17 | 11.88 | Creosol | 3.1 |
| 18 | 12.31 | 2,3-Dihydrobenzofuran | 6.3 |
| 19 | 13.14 | 4-Ethyl-2-methoxyphenol | 5.2 |
| 20 | 13.67 | 4-Hydroxy-3-methoxystyrene | 1.1 |
| 21 | 14.15 | Syringol | 2.6 |
| 22 | 15.53 | Trans-isoeugenol | 1.1 |
| 23 | 16.02 | 3-Hydroxydodecanoic acid | 2.0 |
| 24 | 16.40 | 5-Tert-butylbenzene-1,2,3-triol | 0.7 |

RT: retention time.

**Table S4.** Relative proportion (area%) of most components of CS based bio-oil at 600 W.

| **Entry** | **RT(min)** | **Peak name** | **Area%** |
| --- | --- | --- | --- |
| 1 | 6.04 | 3-Furancarbinol | 1.7 |
| 3 | 8.47 | Phenol | 4.9 |
| 4 | 9.21 | Methyl cyclopentenolone | 1.9 |
| 5 | 9.75 | O-Cresol | 2.5 |
| 6 | 10.11 | p-Cresol | 3.7 |
| 7 | 10.29 | Guaiacol | 4.1 |
| 8 | 10.77 | 3-Ethyl-2-hydroxy-2-cyclopenten-1-one | 1.4 |
| 9 | 11.56 | 4-Ethylphenol | 2.8 |
| 10 | 12.37 | 2,3-Dihydrobenzofuran | 12.2 |
| 11 | 12.98 | Tridecan-2-yl butanoate | 10.4 |
| 12 | 13.20 | 4-Ethyl-2-methoxyphenol | 3.5 |
| 13 | 13.73 | 4-Hydroxy-3-methoxystyrene | 7.4 |
| 14 | 14.20 | Syringol | 4.0 |
| 15 | 16.02 | 2,6,10-Trimethyltetradecane | 1.5 |
| 16 | 16.28 | 2,4-Di-t-butylphenol | 1.0 |
| 17 | 16.45 | 5-Tert-butylbenzene-1,2,3-triol | 0.7 |
| 18 | 18.58 | 2,6,10-Trimethyltetradecane | 2.3 |

RT: retention time.

**Table S5.** Relative proportion (area%) of most components of corn stalk based bio-oil at 800 W.

| **Entry** | **RT (min)** | **Peak name** | **Area%** |
| --- | --- | --- | --- |
| 1 | 6.04 | 3-Furancarbinol | 1.5 |
| 2 | 8.47 | Phenol | 4.7 |
| 3 | 9.21 | Methyl cyclopentenolone | 2.6 |
| 4 | 9.75 | O-Cresol | 3.4 |
| 5 | 10.11 | p-Cresol | 4.6 |
| 6 | 10.29 | Guaiacol | 4.6 |
| 7 | 10.77 | 3-Ethyl-2-hydroxy-2-cyclopenten-1-one | 1.8 |
| 8 | 11.56 | 4-Ethylphenol | 4.5 |
| 9 | 12.37 | 2,3-Dihydrobenzofuran | 10.3 |
| 10 | 12.98 | 4-Ethyl-2-methoxyphenol | 3.9 |
| 11 | 13.20 | 4-Hydroxy-3-methoxystyrene | 6.8 |
| 12 | 13.73 | Syringol | 4.9 |
| 13 | 14.20 | 2-Methoxy-5-prop-1-enylphenol | 1.6 |
| 14 | 16.02 | 2,6,10-Trimethyltetradecane | 2.2 |
| 15 | 16.28 | 2,4-Di-t-butylphenol | 2.9 |
| 16 | 16.45 | 5-Tert-butylbenzene-1,2,3-triol | 1.4 |
| 17 | 18.58 | 2,6,10-Trimethyltetradecane | 3.0 |
| 18 | 18.85 | 3,7,11-Trimethyldodecan-1-ol | 0.8 |

RT: retention time.

**Table S6.** Relative proportion (area%) of most components of corn stalk based bio-oil at 1200 W.

| **No.** | **RT(min)** | **Peak name** | **Area%** |
| --- | --- | --- | --- |
| 1 | 6.06 | 3-Furancarbinol | 0.8 |
| 2 | 8.47 | Phenol | 3.7 |
| 3 | 9.22 | Methyl cyclopentenolone | 2.1 |
| 4 | 9.75 | O-Cresol | 3.2 |
| 5 | 10.12 | p-Cresol | 3.9 |
| 6 | 10.30 | Guaiacol | 4.3 |
| 7 | 10.77 | 3-Ethyl-2-hydroxy-2-cyclopenten-1-one | 1.2 |
| 8 | 11.56 | 4-Ethylphenol | 5.1 |
| 9 | 11.94 | Creosol | 1.1 |
| 10 | 12.36 | 2,3-Dihydrobenzofuran | 10.1 |
| 11 | 13.19 | 4-Ethyl-2-methoxyphenol | 4.0 |
| 12 | 13.72 | 4-Hydroxy-3-methoxystyrene | 7.9 |
| 13 | 14.20 | Syringol | 4.7 |
| 14 | 15.57 | 2-Methoxy-5-prop-1-enylphenol | 1.6 |
| 15 | 16.02 | 2,6,10-Trimethyltetradecane | 2.2 |
| 16 | 16.27 | 2,4-Di-t-butylphenol | 2.7 |
| 17 | 16.44 | 5-Tert-butylbenzene-1,2,3-triol | 1.1 |
| 18 | 18.54 | 2,6,10-Trimethyltetradecane | 2.6 |
| 19 | 18.84 | 3,7,11-Trimethyldodecan-1-ol | 0.7 |

RT: retention time.





**Figure S1.** GC-MS chromatogram of bio-oil produced by microwave pyrolysis of CS at (a)400 ℃, (b) 500 ℃, (c) 600 ℃, (d) 700 ℃.





**Figure S2.** GC-MS chromatogram of bio-oil produced by microwave pyrolysis of CS at (a) 600 W, (b) 800 W, (c) 1000 W, (d) 1200 W.
